# Nitric oxide controls excitatory/inhibitory balance in the hypoglossal nucleus during early postnatal development

**DOI:** 10.1007/s00429-020-02165-9

**Published:** 2020-11-01

**Authors:** Federico Portillo, Bernardo Moreno-López

**Affiliations:** Grupo de Neurodegeneración y Neurorreparación (GRUNEDERE) and Instituto de Investigación e Innovación Biomédica de Cádiz (INiBICA), Área de Fisiología, Facultad de Medicina, Universidad de Cádiz, Plaza Falla 9, 11003 Cádiz, Spain

**Keywords:** Nitric oxide, Synaptic refinement, VGAT, VGLUT2, Myosin light chain, Synaptotoxin, Synaptotrophin

## Abstract

Synaptic remodeling during early postnatal development lies behind neuronal networks refinement and nervous system maturation. In particular, the respiratory system is immature at birth and is subjected to significant postnatal development. In this context, the excitatory/inhibitory balance dramatically changes in the respiratory-related hypoglossal nucleus (HN) during the 3 perinatal weeks. Since, development abnormalities of hypoglossal motor neurons (HMNs) are associated with sudden infant death syndrome and obstructive sleep apnea, deciphering molecular partners behind synaptic remodeling in the HN is of basic and clinical relevance. Interestingly, a transient expression of the neuronal isoform of nitric oxide (NO) synthase (NOS) occurs in HMNs at neonatal stage that disappears before postnatal day 21 (P21). NO, in turn, is a determining factor for synaptic refinement in several physiopathological conditions. Here, intracerebroventricular chronic administration (P7–P21) of the broad spectrum NOS inhibitor l-NAME (*N*(ω)-nitro-l-arginine methyl ester) differentially affected excitatory and inhibitory rearrangement during this neonatal interval in the rat. Whilst l-NAME led to a reduction in the number of excitatory structures, inhibitory synaptic puncta were increased at P21 in comparison to administration of the inactive stereoisomer d-NAME. Finally, l-NAME decreased levels of the phosphorylated form of myosin light chain in the nucleus, which is known to regulate the actomyosin contraction apparatus. These outcomes indicate that physiologically synthesized NO modulates excitatory/inhibitory balance during early postnatal development by acting as an anti-synaptotrophic and/or synaptotoxic factor for inhibitory synapses, and as a synaptotrophin for excitatory ones. The mechanism of action could rely on the modulation of the actomyosin contraction apparatus.

## Introduction

Synaptic remodeling during neonatal development lies behind neuronal networks refinement and nervous system maturation. The adjustment and/or removal of existing synapses and the generation of new synapses are the substratum for synaptic reorganization occurring in learning, memory formation, and refinement of sensorimotor functions (Benson et al. 2001; Sunico et al. 2005). At the neonatal stage, redundant synaptic connections are transiently formed. Once the initial patterns of synaptic connections are settled down, there are a refinement and remodeling of these initial projections into highly tuned and functioning circuits (Goodman and Shatz 1993). During this developmental period, some synapses are strengthened whereas other redundant connections are weakened and eventually eliminated (Kano and Watanabe 2019). Up to date, experimental models to study postnatal synapse remodeling include structures such as the cerebellum and the dorsal lateral geniculate nucleus (Kano and Watanabe 2019). Alternatively, respiratory-related structures are also long being used to investigate mechanisms underlying postnatal networking and functional maturation. In this context, respiratory-related motor neurons, mainly hypoglossal motor neurons (HMNs), have been the object of study focused on changes in morphology, membrane receptors, synaptic array and/or electrophysiological properties lying behind functional and structural maturation (Marchetti et al. 2002; Greer and Funk 2005; Carrascal et al. 2015; Kanjhan et al. 2016b).

Respiration is critical during development, throughout life, and must generate reliable, rhythmic motor output through an elaborate system of control (Williams et al. 2019a). However, the respiratory system is immature at birth and significant postnatal development occurs (Feldman et al. 2013). Thus, autonomic regions in the brainstem, which generate and control respiratory rhythm, are subjected to perinatal refinement for correct maturation. The normal functioning of the CNS is maintained by a fine-tuned balance between excitation and inhibition (Mody et al. 1994). The second postnatal week is a critical period in respiratory network development in the rat, when abrupt neurochemical, metabolic, and physiological changes are evident. In this context, a sudden drop in expression of excitatory and heightened expression of inhibitory neurochemicals at P12–P13 occur in multiple respiratory-related brainstem nuclear groups of the rat including the hypoglossal motor nucleus (HN) (Gao et al. 2011). HMNs innervate the tongue and pharyngeal muscles supporting a number of vital functions such as respiration, chewing, sucking, swallowing, and phonation. In the transition from the neonatal (P5–P8) to the adult stage, rat HMNs suffer a reduction of ~ 30% of the synaptic coverage (Sunico et al. 2005, 2010). Nevertheless, total number of synapses and number of excitatory and inhibitory boutons in the HN increased from birth to P20, followed by a decrease in adults (O’Kusky 1998). Strikingly, an excitatory/inhibitory imbalance in HMNs occurs during the critical period of postnatal development (P12–P13) (Gao et al. 2011). Given that, abnormalities in the normal development of HMNs have been associated with sudden infant death syndrome (Konrat et al. 1992; Lavezzi et al. 2010) and obstructive sleep apnea (Remmers et al. 1978), deciphering molecular partners behind synaptic remodeling in the HN is also of clinical relevance.

A firm candidate to impact on excitatory/inhibitory refinement in the HN during postnatal maturation is the free radical nitric oxide (NO), which is synthesized by NO synthase (NOS). NO is a three-dimensional-acting diffusible factor “necessary” and “sufficient” for synaptic stripping of adult HMNs in pathological conditions (Sunico et al. 2005, 2010; Moreno-López and González-Forero 2006; Montero et al. 2010; Moreno-López et al. 2011; González-Forero and Moreno-López 2014). At pathological concentrations, NO acts as a synaptotoxic agent evoking withdrawal of stable synapses and avoiding formation of new synaptic contacts on injured HMNs (Sunico et al. 2005; Moreno-López and González-Forero 2006). Pathological NO at neonatal stage induces loss of both excitatory and inhibitory boutons even though excitatory was more profoundly affected than inhibitory synapses. But, remarkably, NO only affects excitatory inputs in adult rats (Sunico et al. 2010; Moreno-López et al. 2011; González-Forero and Moreno-López 2014). Mechanism of action by which NO triggers synaptic loss involves increased presynaptic myosin light chain phosphorylation (pMLC) preceding synapse destabilization (Sunico et al. 2010; Moreno-López et al. 2011). It is well known that pMLC triggers actomyosin contraction and neurite retraction (Luo 2000, 2002; Etienne-Manneville and Hall 2002). Nevertheless, the role of physiological NO in synaptic refinement of HMNs during postnatal development remains elusive. Although, adult motor neurons lack in NOS (Moreno-López 2010), it is interesting to remark that transient expression of the neuronal isoform of NOS (NOS-I) occurs in HMNs at neonatal stage that fully disappears before P21 (Vazquez et al. 1999). In this way, it has been postulated that under physiological nanomolar concentrations of NO, probably reached during postnatal maturation, a preferential loss of NO-sensitive inhibitory synapses might occur (Sunico et al. 2010).

Here, we hypothesize that endogenous NO determines excitatory/inhibitory balance in the HN during early postnatal maturation. Thus, chronic administration of a NOS inhibitor (*N*(ω)-nitro-l-arginine methyl ester, l-NAME) differentially affected excitatory and inhibitory inputs, then, altering excitatory/inhibitory ratio in the HN. Furthermore, l-NAME induced a reduction in the levels of pMLC in the nucleus, suggesting that mechanism by which endogenous NO regulates synaptic refinement could involve the actomyosin contraction apparatus.

## Materials and methods

### Animals

Neonatal (P5) Wistar rats of either sex were obtained from an authorized supplier (Animal Supply Services, University of Cádiz, Cádiz, Spain). Animal care and handling followed the guidelines of the European Union Council (2010/63/EU, 86/609/UE) on the use of laboratory animals. Experimental procedures were approved by the local Animal Care and Ethics Committee (University of Cádiz, Cádiz, Spain) and the Ministry of Agriculture, Fisheries and Rural Development (Junta de Andalucía, Spain). Experimental animals were individually housed with their mother in cages with water and food pellets available ad libitum, at 21 ± 1 °C, with a 12-h light/dark cycle. Efforts were made to minimize the number of animals used and their suffering. All surgical processes were carried out under aseptic conditions. At least three animals per experimental condition were used. Data obtained from 27 pups were used for this study.

### Drug administration

**Fig. 1:**
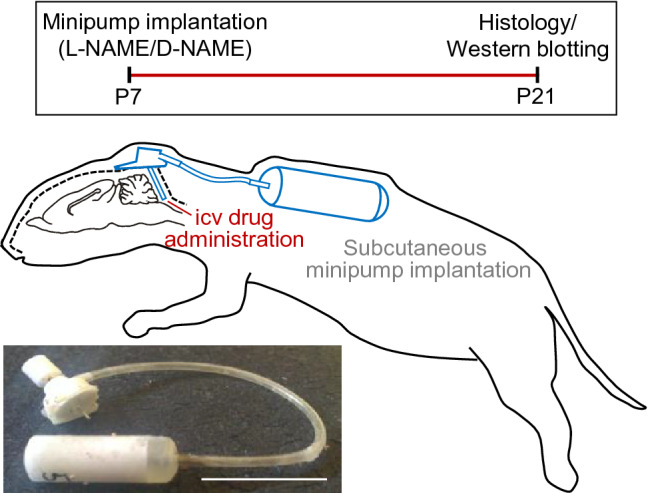
Schematic illustrating the experimental protocol. An osmotic minipump, filled with l-NAME or d-NAME, was subcutaneously implanted and connected via a catheter to an infusion cannula fixed to the skull to deliver the drug into the fourth ventricle. Minipump implantation was performed at P7 and tissue extraction for histological processing or western blotting analysis was performed at P21. Bottom, example of osmotic minipump connected to the infusion system extracted from an experimental animal. Minipump was then weighed to assure drug infusion. Scale bar, 10 mm

After habituation for 48 h, the infusion system was implanted at P7 and, subsequently, pups were housed with their respective mother. Only animals which appropriately recovered after surgery were included in the study (27 of 35, ~ 75%). For drug delivery into the fourth ventricle a stainless steel brain infusion cannula (30 gauge) was chronically implanted and connected by a Vinyl catheter (Alzet Brain infusion kit 3) to a osmotic minipump (volume: 100 μl, delivery rate: 0.25 μl/h; Alzet 1002, Durect, Cupertino, CA). Anesthetized (1.5–3% isoflurane mixed with 100% O_2_) animals received intramuscularly atropine (0.2 mg/kg) and dexamethasone sodium phosphate (0.8 mg/kg). The absence of withdrawal reflexes was considered a signal of sufficient deep anesthesia. Subsequently, pups were fixed with appropriated adaptors in a stereotaxic frame and the skin was incised longitudinally after disinfecting. The infusion cannula was carefully advanced throughout a drilled round window (0.5 mm in diameter) in the midline of the interparietal–occipital bones junction. Cannula was placed in parallel and close to the inner portion of the occipital bone (Fig. 1). The infusion system was finally attached to the skull by means of an instant adhesive gel (Loctite 454, Alzet) once the end of infusion cannula was positioned near to the fourth ventricle as previously performed (García-Morales et al. 2015, 2019). Finally, a small incision was made at the base of the neck and stretched with a hemostat to facilitate implantation. The osmotic minipump was then placed subcutaneously through the opening. Afterward, all incisions were sutured with Histoacryl^®^ (Braun Surgical SA, Barcelona, Spain). All animals received one post-operative injection of penicillin (20,000 i.u./kg; i.m.) to prevent infection. Pyrazolone (0.1 mg/kg; i.m.) was given on awakening for post-operative analgesia. Drugs (l-/d-NAME) were dissolved in sterile saline and their concentration were adjusted to assure a chronic delivery of 180 mg/kg/day for 2 weeks.

### Immunohistochemistry

Obtaining samples and immunohistochemistry processing have been performed as previously described with minimal differences (García-Morales et al. 2015, 2019). Juvenile (P21) rats were anesthetized with chloral hydrate (0.5 g/kg; i.p.), injected intraventricularly with heparin, and perfused transcardially first with phosphate buffer (PB) saline (PBS), followed by 4% paraformaldehyde in 0.1 M PB, pH 7.4, at 4 °C. The brains were removed, postfixed for 2 h in the same fixative solution, and cryoprotected by overnight immersion in 30% sucrose in PB at 4 °C. Serial coronal sections (30-μm thick) from brainstem were obtained using a cryostat and stored at − 20 °C in a cryoprotectant solution (glycerol and PBS, pH 7.4, 1:1 in volume) for latter immunolabeling.

Immunohistochemistry was performed against the vesicular glutamate transporter 2 (VGLUT2), the vesicular GABA/glycine transporter (VGAT), to identified excitatory and inhibitory structures, respectively, and the non-phosphorylated form of neurofilament H (SMI32) as a motor neuron marker. Sections were rinsed in PBS and immersed in 2.5% (w/v) bovine serum albumin, 0.25% (w/v) sodium azide, and 0.1% (v/v) Triton X-100 in PBS for 30 min, followed by overnight incubation at 4 °C with the combination of VGLUT2/SMI32 or VGAT/SMI32 antisera. Polyclonal primary antibodies anti-VGAT (1:2000, Millipore Bioscience Research Reagents, Cat# AB5062P, RRID:AB_2301998), developed in rabbit; anti-VGLUT2 (1:2000, Millipore Bioscience Research Reagents, Cat# AB2251, RRID:AB_1587626) developed in guinea pig; and monoclonal primary antibody anti-SMI32 (1:8000, Covance Research Products Inc, Cat# SMI-32R-500, RRID:AB_509998) developed in mouse were used in this study. Subsequently, the tissue was rinsed 3 times with PBS for 5 min each and incubated for 2 h at room temperature with the secondary antibodies, developed in donkey: anti-rabbit, anti-guinea pig, and anti-mouse IgGs labeled with the cyanine 2, 3, or 5 (Cy2, Cy3, Cy5) (1:400; Jackson ImmunoResearch Laboratories). Finally, sections were washed with PBS and mounted on slides with a solution containing propyl gallate (0.1 mM in PBS/glycerol, 1:9 v/v). Animals and tissue were processed in parallel.

**Fig. 2:**
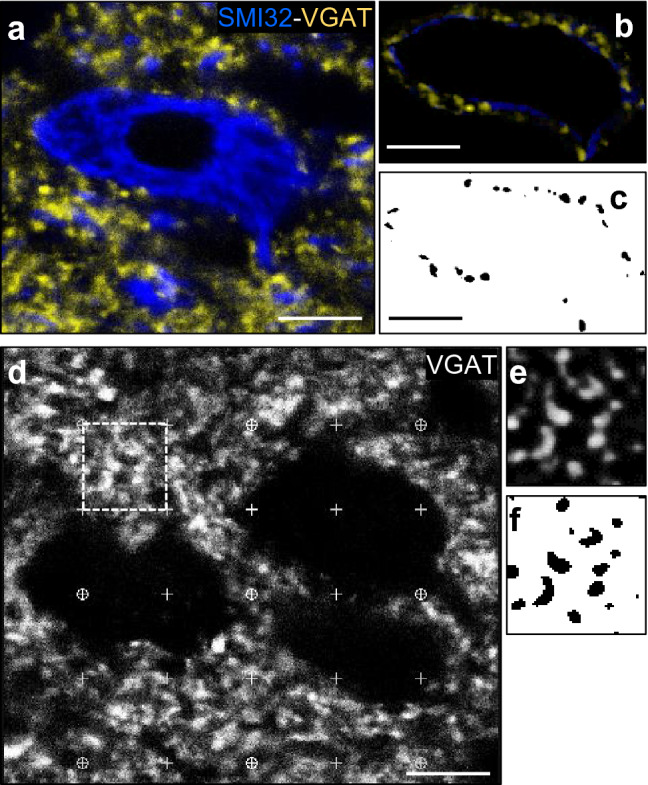
Quantitative estimation of immunoreactive synaptic puncta. **a** High magnification confocal image of HN immunolabeled for VGAT (yellow) and the motoneuron marker SMI32 (blue). **b**, **c** After background subtraction, VGAT-ir puncta were manually isolated and smoothed (**b**) to delineate puncta using ImageJ software. Finally, images were converted into binary images (**c**). **d** As in **a**, but images was converted to a greyscale. Channel for SMI32 immunolabeling was omitted to analyze synaptic puncta. Grid composed by squares of 100 μm^2^ used to quantify puncta density in neuropil using randomly sampled images is overimposed. Boxed area illustrates an analyzed region of interest. **e, f** As in** b**,** c**, for the boxed area in** d**. The size and number of dots in binary images were stored for posterior statistical analysis. Fluorescence intensity for each dot was measured from the original image in** a** or** d**. See “Materials and methods” for more details. Scale bar, 10 μm

Sections were analyzed using an Olympus FV1000-MPE confocal microscope (Olympus, Japan). Omission of the primary antibodies resulted in no detectable staining. Images for quantification were acquired under a 40 × oil-immersion objective through a *z*-plane in which maximum antibody penetration was evidenced. For multichannel image acquisition, fluorophores were excited alternately. Pinholes were set to the same airy unit for each laser to obtain identical optical section thickness. For comparison between different experimental conditions, images were acquired using the same settings. The perimeter, density, fluorescent intensity, and dimensions of immunolabeled structures were assessed with ImageJ 1.48 v (NIH). Only HMN somata at the level of the nucleus were included in the study. Images for quantification were flattened and background-filtered to enhance cluster outlines and an user-defined intensity threshold was applied to select puncta as previously described (Bannai et al. 2009; García-Morales et al. 2015). Briefly, to delineate synaptic structures, VGAT or VGLUT2 channel was first processed for background subtraction to obtain the maximum dynamic range of intensities (from 0 to 250). Next, images were subjected to a smoothing filter (same for all) and subsequently were binarized based on local differences in intensity (Fig. 2). Only puncta reporting an area of > 0.04 μm^2^ were taken as specific. Synaptic puncta apposed to HMNs somata and in the neuropil were differentially analyzed. Immunoreactive-puncta apposed to somata are presented as the number of synaptic structures per HMN and as linear density (puncta/100 μm of HMN perimeter). On the other hand, a grid composed by 16 squares (10 μm × 10 μm) was randomly distributed along the HN in each section to characterize puncta in the neuropil. Only those synaptic structures included in squares that did not contain somata were analyzed (Fig. 2d). Area density (puncta/μm^2^) in neuropil was compared between treatments. Treatments were blinded for researcher who performed measures.

### Western blotting

Tissue extraction and western blotting have been performed as previously described (Sunico et al. 2010). Rats (P7, P10, P18, and P21), untreated or treated with d-LAME or l-NAME (Fig. 1), were anesthetized by hypothermia (placing on ice for 10–15 min) and decapitated, and their brainstems quickly removed. Dissection was in ice-cold (< 4 °C) sucrose artificial CSF (S-aCSF) bubbled with 95% O_2_ and 5% CO_2_. S-aCSF composition was as follows (in mM): 26 NaHCO_3_, 10 glucose, 3 KCl, 1.25 NaH_2_PO_4_, 2 MgCl_2_, and 218 sucrose. Slicing and microdissection of HNs were both performed in ice-cold (< 4 °C) S-aCSF supplemented with protease (1 mM phenylmethylsulfonyl fluoride, 10 mg/ml leupeptin, 10 mg/ml pepstatin A, and 10 mg/ml aprotinin) and phosphatase inhibitors. Transverse slices (300–400-μm thick) were obtained using a vibroslicer (NVSL; WPI). Microdissected HNs were homogenized in lysis buffer [50 mM Tris/HCl, pH 7.4, 1% (v/v) Triton X-100, 0.5% (w/v) sodium deoxycholate] supplemented with protease and phosphatase inhibitors using a 1-ml syringe. Equal amounts of protein were processed for SDS-PAGE and immunoblotting, using a specific antibody against pMLC (1:200; Santa Cruz Biotechnology, Cat# sc-17557, RRID:AB_670127) and MLC (1:200; Santa Cruz Biotechnology, Cat# sc-25618, RRID:AB_649811) developed in goat and rabbit, respectively. Membranes were also probed with anti-α_1_-tubulin antibody (1:250,000; Sigma-Aldrich, Cat# T9026, RRID:AB_477593) developed in mouse, as a control for the total amount of protein contained in each well.

### Statistics

Data are expressed as mean ± SEM. Unpaired two-tailed Student’s *t* or Kolmogorov–Smirnov tests were used to compare data or frequency distributions between two groups, respectively. When *n *≤ 5, as in western blotting studies, the non-parametric Mann–Whitney *U* test was applied.

## Results

### l-NAME impacts on inhibitory refinement

**Fig. 3:**
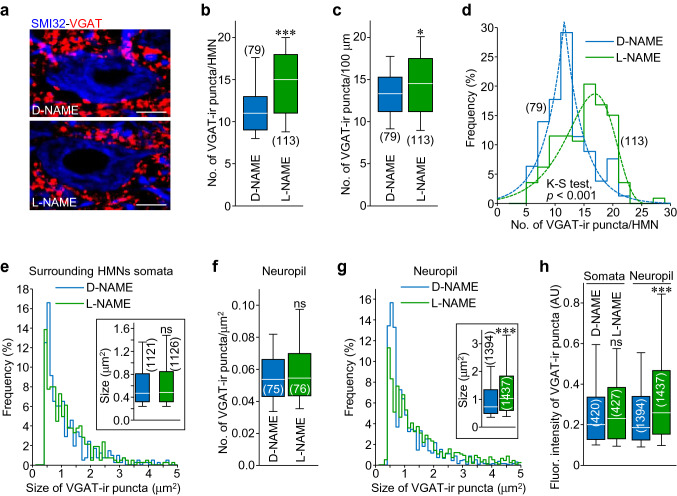
l-NAME-treatment increases inhibitory puncta apposed to HMNs somata. **a** High magnification confocal images showing two SMI32-ir HMNs (blue) and surrounding VGAT-positive puncta (red) from P21 rats treated with d-NAME (top) or l-NAME (bottom). Scale bars, 10 μm.** b**,** c** Number of VGAT-ir puncta per HMN (**b**) and per 100 μm of somata perimeter (**c**) after d-NAME (blue) or l-NAME (green) treatment. **d** Frequency histogram of the number of VGAT-ir puncta per HMN receiving indicated treatments. Bin, 2 puncta/HMN. Dashed lines represent Gaussian curve fits for each treatment (d-NAME, *r*^2^ = 0.93; l-NAME, *r*^2^ = 0.89). Statistic output for Kolmogorov–Smirnov test between the two distributions is stated in the plot. **e** Frequency histogram of the size of VGAT-ir puncta apposed to HMNs somata under indicated treatments. Bin, 0.1 μm^2^. Inset, box plot of puncta size after d-NAME or l-NAME treatment. **f** Number of VGAT-ir puncta per μm^2^ in the neuropil after stated treatments. **g** As in **e** but for puncta analyzed in the neuropil. **h** Fluorescence (Fluor.) intensity of analyzed VGAT-ir puncta apposed to HMNs and in the neuropil after d-NAME or l-NAME-treatment. Box plots show median (white line) and the 25–75% range as box, the whiskers indicate 5–95% range. The number of analyzed HMNs (**b**–**d**), regions of interest in the neuropil (**f**) or puncta (**e**, **g**, **h**) are presented in parentheses. *p < 0.05, **p < 0.01, ***p < 0.001; *n.s.* not significant; by Student *t* test

To assess the impact of endogenous NO on synaptic refinement in the HN during early postnatal development, the broad-spectrum NOS inhibitor l-NAME (180 mg/kg/day) was chronically infused in the fourth ventricle from P7 to P21 by means of an osmotic minipump (Fig. 1). Immunohistochemistry against the vesicular GABA/glycine transporter (VGAT) was performed to identify synaptic vesicle pools at inhibitory inputs, then it was used as a inhibitory synaptic marker. At P21, l-NAME-treated rats displayed an increase in both, the number (+ 24.4 ± 3.5%) and linear density (+ 9.2 ± 2.9%) of inhibitory puncta (VGAT-ir) apposed to HMNs somata, in comparison with animals which received the inactive stereoisomer d-NAME (Fig. 3a–d, Table 1). However, the size of VGAT-ir structures remains unaltered by the NOS inhibitor (Fig. 3e, Table 1). Interestingly, the reciprocal was observed at the neuropil. Whilst l-NAME did not impact on density of inhibitory puncta, VGAT-ir vesicle pools were larger (+ 38.0 ± 3.7%) than after d-NAME-treatment (Fig. 3f, g, Table 1). Finally, fluorescence intensity of VGAT-ir structures was only affected (+ 38.2 ± 3.3%) by NOS inhibitor in puncta located at the neuropil but not in those around HMN somata (Fig. 3h). In summary, a net increase of the inhibitory influence in the HN was noted at P21 after l-NAME-treatment. This alteration was accounted by an increase in the number of inhibitory puncta around HMN perikarya together with an enhancement of the size of vesicle pools in the neuropil. These outcomes suggest that endogenous NO is unfavorable for some inhibitory inputs in the HN during postnatal maturation.

**Table 1:** VGAT-ir puncta in the HN

Treatment	Somatic puncta			Neuropil puncta	
	Number^a^	Linear density^b^	Size (μm^2^)	Area density^c^	Size (μm^2^)
d-NAME	11.71 ± 0.42	13.37 ± 0.38	0.71 ± 0.02	0.057 ± 0.002	1.08 ± 0.03
l-NAME	14.57 ± 0.41***	14.60 ± 0.39*	0.71 ± 0.02	0.060 ± 0.003	1.49 ± 0.04***

*p < 0.05, **p < 0.01, ***p < 0.001 by Student *t* test

^a^Number of puncta per HMN somata, ^b^number of puncta per 100 μm, ^c^number of puncta per μm^2^

### l-NAME affects excitatory rearrangement

**Fig. 4:**
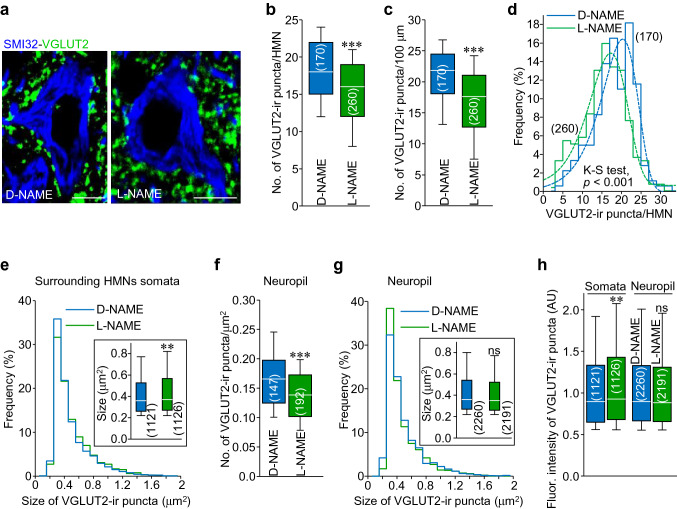
l-NAME-treatment reduces excitatory puncta in the HN **a** High magnification confocal images showing two SMI32-ir HMNs (blue) and surrounding VGLUT2-positive puncta (green) from P21 rats treated with d-NAME (left) or l-NAME (right). Scale bars, 10 μm. **b, c** Number of VGLUT2-ir puncta per HMN **b** and per 100 μm of somata perimeter **c** after d-NAME (blue) or l-NAME (green) treatment. **d** Frequency histogram of the number of VGLUT2-ir puncta per HMN receiving indicated treatments. Bin, 2 puncta/HMN. Dashed lines represent Gaussian curve fits for each treatment (d-NAME, *r*^2^ = 0.90; l-NAME, *r*^2^ = 0.95). Statistic output for Kolmogorov–Smirnov test between the two distributions is stated in the plot. **e** Frequency histogram of the size of excitatory puncta apposed to HMNs somata under indicated treatments. Bin, 0.1 μm^2^. Inset, box plot of puncta size after d-NAME or l-NAME-treatment. **f** Number of VGLUT2-ir puncta per μm^2^ in the neuropil after stated treatments. **g** As in **e** but for puncta analyzed in the neuropil. **h** Fluorescence (Fluor.) intensity of analyzed VGLUT2-ir puncta apposed to HMNs and in the neuropil after d-NAME- or l-NAME-treatment. Box plots show median (white line) and the 25–75% range as box, the whiskers indicate 5–95% range. The number of analyzed HMNs (**b**–**d**), regions of interest in the neuropil (**f**) or puncta (**e**,** g**,** h**) are presented in parentheses. *p < 0.05, **p < 0.01, ***p < 0.001; *n.s.* not significant; by Student *t* test

Immunohistochemistry against the vesicular glutamate transporter 2 (VGLUT2) was performed to label a subtype of excitatory synaptic inputs. In contrast to what happened with inhibitory puncta, l-NAME-treatment induced a reduction in both, the number (− 15.9 ± 1.7%) and linear density (− 19.6 ± 1.7%) of VGLUT2-ir puncta apposed to HMN somata, in comparison with animals which received the inactive stereoisomer d-NAME (Fig. 4a–d, Table 2). Interestingly, the size of excitatory vesicle pools was increased (+ 4.5 ± 1.8%) by the NOS inhibitor (Fig. 4e, Table 2). At the neuropil, l-NAME affected density (− 17.6 ± 2.4%), but not size, of VGLUT2-ir structures (Fig. 4f, g, Table 2). Finally, fluorescence intensity of excitatory puncta was only affected (+ 6.7 ± 1.9%) by l-NAME in synaptic vesicle pools around HMNs somata (Fig. 4h). Thus, a reduction in the excitatory signaling in the HN was noted at P21 after l-NAME-treatment. This alteration was accounted by a decline in the number of excitatory puncta around HMN perikarya, although a partial compensatory effect might be expected by increase in the synaptic vesicle pool size, together with a decrease in puncta density in the neuropil. Altogether, results support that physiologically synthesized NO has a positive impact on certain excitatory inputs in the HN during postnatal maturation.

**Table 2:** VGLUT2-ir puncta in the HN

Treatment	Somatic puncta			Neuropil puncta	
	Number^a^	Linear density^b^	Size (μm^2^)	Area density^c^	Size (μm^2^)
d-NAME	18.2 ± 0.33	20.9 ± 0.39	0.44 ± 0.007	0.17 ± 0.005	0.45 ± 0.006
l-NAME	15.3 ± 0.31***	16.8 ± 0.35***	0.46 ± 0.008**	0.14 ± 0.004***	0.44 ± 0.006

*p < 0.05, **p < 0.01, ***p < 0.001 by Student *t* test

^a^Number of puncta per HMN somata, ^b^number of puncta per 100 μm, ^c^number of puncta per μm^2^

### l-NAME-induced alterations in excitatory/inhibitory balance

Next, we analyzed the effect of the NOS inhibitor on the excitatory/inhibitory ratio in the HN. After 2-week treatment with l-NAME, the excitatory/inhibitory balance was strongly altered in comparison with ratio observed in d-NAME-treated animals (Table 3). Whilst the number (+ 55.4 ± 2.8%) and linear density (+ 56.3 ± 2.9%) of VGLUT2-ir puncta were higher than VGAT-ir ones apposed to HMNs in d-NAME-treated rats, this ratio was strongly reduced (number: + 5.0 ± 2.1%; linear density: + 15.1 ± 2.4%) under l-NAME-treatment (Table 3). Given that, the mean size of excitatory structures near perikarya was increased by the NOS inhibitor (Fig. 4e, Table 2), one possibility arises that size increase in synaptic vesicle pools at excitatory inputs could compensate in some degree l-NAME-induced alterations in excitatory/inhibitory balance on HMNs by increasing the number of disposable synaptic vesicles. In this context, the mean total area of VGLUT2-ir and VGAT-ir puncta was the result of the multiplication of the mean size of synaptic structures by the mean number of puncta per HMN (Table 3). Thus, the ratio of total area of VGLUT2/VGAT puncta was 0.96 in d-NAME versus 0.68 in l-NAME-treated animals, which reports a reduction of ~ 30% after application of the NOS inhibitor (Table 3). At the neuropil, the density of excitatory versus inhibitory structures were robustly reduced after l-NAME- (+ 133.3 ± 0.7%) relative to d-NAME-treatment (+ 198.0 ± 0.9%) (Table 3). Furthermore, l-NAME induced a decrease (~ − 45%) of the area occupied by VGLUT2-ir in relation to VGAT-ir puncta (Table 3). This agrees with reduction observed in the density of excitatory structures (Fig. 4f, Table 2) in parallel to increase in the size of inhibitory ones (Fig. 3g, Table 1) induced by the NOS inhibitor. Taken together, outcomes agree with the hypothesis that endogenous NO controls excitatory/inhibitory balance by promoting maintenance and/or gain of glutamatergic inputs and destabilization of GABA/glycinergic synapses during early postnatal maturation of the HN.

**Table 3:** VGLUT2/VGAT ratio in the HN

Treatment	Somatic puncta			Neuropil puncta	
	Number^a^	Linear density^b^	Area/HMN^c^	Area density^d^	Area/μm^2e^
d-NAME	1.6 (18.2/11.7)	1.6 (20.9/13.4)	0.96 (8.0/8.3)	3.0 (0.17/0.057)	1.3 (0.08/0.06)
l-NAME	1.1 (15.3/14.6)	1.2 (16.8/14.6)	0.68 (7.0/10.3)	2.3 (0.14/0.060)	0.7 (0.06/0.09)

Ratio and mean values (in parentheses) for VGLUT2/VGAT-ir puncta

^a^Number of puncta per HMN somata, ^b^number of puncta per 100 μm, ^c,e^ratio of the total area of puncta per HMN somata^c^ or per μm^2e^, ^d^number of puncta per 100 μm^2^, ^e^values in parentheses are in μm^2^

### l-NAME alters the size of HMNs somata

When the number of synaptic puncta was expressed relative to motor neuron size, l-NAME-induced linear density alterations were attenuated for inhibitory puncta or strengthened for excitatory ones in comparison to absolute number of synaptic structures expressed per HMN. That is, whereas the number of VGAT-ir puncta increased in ~ 25% under l-NAME-treatment, linear density only changed in ~ 9% (Fig. 3b, c, Table 1). On the other hand, NOS inhibitor-induced reduction in the number of excitatory structures around HMNs (~ − 16%) was accentuated (~ − 20%) when relativized to perykaria perimeter (Fig. 4b, c, Table 2). These observations argued on a presumably effect of l-NAME on somata size. At this point, it is interesting to remark that HMNs somata size experienced an increase of approximately 40% from P1 to P21 (Williams et al. 2019a, b). Thus, we hypothesized that l-NAME effects HMNs development by impacting on normal morphological changes, including adjustment in somata size, during postnatal development.

**Fig. 5:**
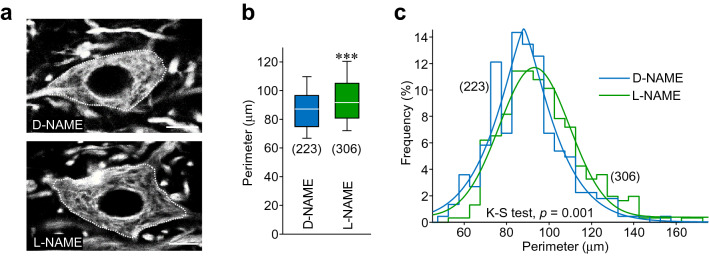
l-NAME-treatment impacts on HMNs somata perimeter. **a** High magnification confocal images showing two SMI32-ir HMNs from P21 rats treated with d-NAME (top) or l-NAME (bottom). Dotted lines indicate the measured perimeter per each HMN. Scale bars, 10 μm. **b** Box plot of the perimeter of analyzed HMNs after d-NAME (blue) or l-NAME (green) treatment. Box plot shows median (white line) and the 25–75% range as box, the whiskers indicate 5–95% range. **c** Frequency histogram of the HMNs somata perimeter after receiving indicated treatments. Bin, 5 μm. Solid lines represent Gaussian curve fits for each treatment (d-NAME, *r*^2^ = 0.92; l-NAME, *r*^2^ = 0.96). Statistic output for Kolmogorov–Smirnov test between the two distributions is stated in the plot. The number of analyzed HMNs (**b**, **c**) are presented in parentheses. *p < 0.05, **p < 0.01, ***p < 0.001; *n.s.* not significant; by Student *t* test

In this way, the somata perimeter of SMI32-immunolabeled HMNs was measured at P21 in d-NAME- and l-NAME-treated rats. Strikingly, the mean perimeter of HMNs perykaria after d-NAME-treatment (87.5 ± 1.2 μm) was lower than after administration of the NOS inhibitor (94.5 ± 1.1 μm) (Fig. 5a, b). This involved a displacement to the right in the frequency distribution of the HMNs somata perimeter (Fig. 5c). Therefore, these outcomes support that endogenous NO not only regulates excitatory/inhibitory balance in the HN, but also control morphological changes of HMNs during postnatal development.

### l-NAME reduces pMLC levels in the HN

**Fig. 6:**
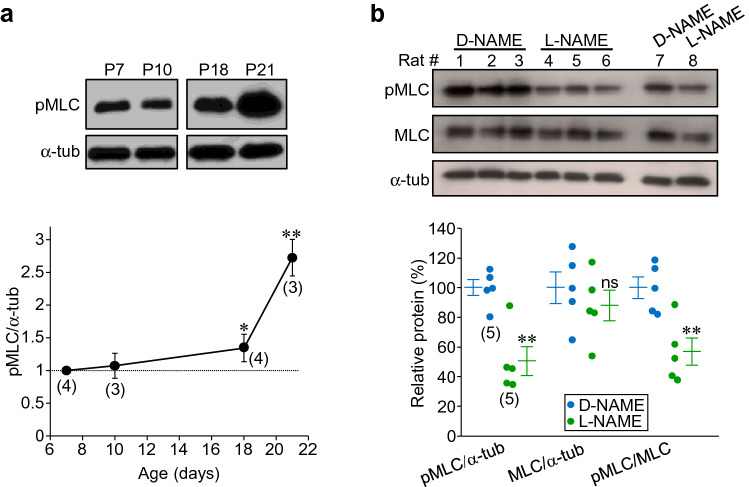
l-NAME reduces pMLC levels in the HN during early postnatal development. **a** Western blots (top) and plot showing pMLC levels, relativized to α-tubulin (α-tub) taken as an internal loading reference, in microdissected HNs from rats at the indicated ages after birth. Values obtained at P7 was taken as 1. **b** Western blots (top) and plot of pMLC, MLC, and α-tub showing that pMLC, but not MLC, levels at P21 were reduced under l-NAME- in comparison to d-NAME-treatment. Four rats per treatment are shown in immunoblots. Mean values obtained for d-NAME was taken as 100%. Number of animals per time point or treatment are indicated in parentheses. Symbols indicate values obtained from each animal. Mean ± SEM are also shown in the plots. *p < 0.05, **p < 0.01, *n.s.* not significant; non-parametric Mann–Whitney *U* test

It is well known that pMLC triggers actomyosin contraction and neurite retraction (Luo 2000, 2002; Etienne-Manneville and Hall 2002). In this context, we recently proposed that pathological NO triggers synaptic loss by promoting MLC phosphorylation at the presynaptic counterpart preceding synapse destabilization (Sunico et al. 2010; Moreno-López et al. 2011). Thus, we look for evidence that the mechanism of action by which NO controls synaptic refinement in the HN during postnatal development involves pMLC, as an indirect indicator of actomyosin contraction. First, we studied the levels of pMLC at different time points of interest during early perinatal progress. Whilst pMLC levels in microdissected HNs were kept stable at P7 (100 ± 0.0%) and P10 (107.3 ± 19.2%), a significant increase in the phosphorylated form of MLC was observed at P18 (134.3 ± 20.8%) and P21 (272.8 ± 28.1%) (Fig. 6A). Finally, at P21, l-NAME-treatment from P7 to P21, reduced pMLC levels as relativized to α-tub (− 49.5 ± 9.7%) and MLC (− 47.2 ± 9.2%), although did not alter total amount of MLC (− 12.1 ± 10.4%) in the HN, in comparison to d-NAME-treated rats (Fig. 6b). Therefore, these results open the possibility that control by NO of synaptic maturation during early postnatal development could involve, at least in part, activity regulation of the actomyosin contraction apparatus.

## Discussion

We report here that endogenous NO tunes excitatory/inhibitory balance in the HN in the P7–P21 early postnatal interval. Synthesized NO during postnatal development seems to act as an anti-synaptotrophic factor or a synaptotoxin for certain inhibitory synapses, whilst it favors stabilization and/or gain of some excitatory inputs thus supporting a synaptotrophic action of NO on this type of synapses. Furthermore, NO impacts on morphological maturation of HMNs during the neonatal stage by regulating, at least, somata size. The mechanism of action by which endogenous NO regulates postnatal maturation of the HN could involve MLC phosphorylation and subsequent modulation of the actomyosin contraction apparatus. Since deviations in the normal development of HMNs have been related with sudden infant death syndrome (Konrat et al. 1992; Lavezzi et al. 2010) and obstructive sleep apnea (Remmers et al. 1978), our data suggest that alterations in NOS expression and/or NO synthesis during this critical period could lie behind both pathological conditions.

Previous studies find a net reduction of inhibitory inputs incoming to HMNs in the transition interval from neonatal to adult stages (Sunico et al. 2010; Paik et al. 2019). On the contrary, the quantity of asymmetric, putative excitatory, synapses increases with differentiation up to P20 (Davidoff and Galabov 1975; Paik et al. 2019). Interestingly, postnatal synaptic refinement in the HN consists in progressive and regressive phases of synaptogenesis involving an initial increase in density and total number for asymmetric and symmetric synapses from birth to P20, followed by a significant decrease of both in adults (O’Kusky 1998). Consistently, here, d-NAME-treated rats showed an increase in the linear density of somatic VGLUT2-ir puncta at P21 (20.9 ± 0.39/100 μm) as compared with that we obtained from untreated HMNs at P7 (6.6 ± 0.49/100 μm) (Sunico et al. 2010). However, d-NAME-treated animals displayed at P21 a reduction, rather than an increase, in the linear density of somatic inhibitory puncta (13.26 ± 0.34/100 μm) relative to that we previously reported at P7 (19.9 ± 0.8/100 μm) (Sunico et al. 2010). The reason for this apparent discrepancy may lie behind different analytical procedures performed, i.e. electron vs confocal microscopy. In this line of argument, single VGAT-ir puncta does not necessarily correspond to an individual synaptic bouton because of VGAT-ir vesicles pools from adjacent boutons could be considered as a unique puncta under confocal analysis.

Many molecules are involved in synaptogenic events during nervous system maturation (Benson et al. 2001; Garner et al. 2002; Yamagata et al. 2003; Stevens et al. 2007). NO is a candidate diffusible molecule linking spatiotemporally events that occur in presynaptic and postsynaptic structures to refine synaptic contacts. NO participates in projection refinement during development (Wu et al. 1994) and in synapse loss suffered by HMNs after motor nerve injury and in an animal model of amyotrophic lateral sclerosis (ALS) (Sunico et al. 2005, 2010, 2011; Moreno-López and González-Forero 2006; Montero et al. 2010; Moreno-López et al. 2011). In the opposite direction, NO promotes formation of new synapses in developing olfactory receptor neurons (Roskams et al. 1994) and in hippocampal organotypic slice cultures (Nikonenko et al. 2003). Furthermore, transient expression of NOS-I occurs in HMNs at neonatal stage that fully disappears before P21 (Vazquez et al. 1999). Accordingly, chronic treatment with the NOS inhibitor l-NAME resulted in a reduction in the number and density of excitatory puncta around HMNs somata and in the neuropil but, in contrast, caused an increase in inhibitory puncta apposed to HMNs perikarya at P21. These outcomes are consistent with a synaptotrophic action of NO on excitatory puncta by promoting net stabilization of excitatory puncta and/or formation of new synaptic structures, but, in contrast, NO seems to act as an anti-synaptotrophic or synaptotoxic factor for VGAT-ir puncta favoring synaptic disruption and/or loss. In this way, pathological concentrations of NO induced a reduction in both VGAT-ir and VGLUT2-ir puncta around neonatal HMNs which actually represented a loss of inhibitory and excitatory boutons from HMNs even though excitatory were more profoundly affected than inhibitory synapses (Sunico et al. 2010). Strikingly, pathologically induced NOS-I expression in adult HMNs led to a NO-triggered and selective loss of excitatory inputs (Sunico et al. 2010). Therefore, results obtained in the present work support our preceding hypothesis that under physiological concentrations of NO, as that probably reached during postnatal maturation, a predominant loss of NO-sensitive inhibitory synapses could occur throughout perinatal development (Sunico et al. 2010).

The source of NO-controlling synaptic refinement during this interval is likely the transient expression of NOS-I in HMNs occurring during this critical period (Vazquez et al. 1999), although another different origins cannot be fully discarded. This idea is strengthened by several findings: (1) it is well known that adult motor neurons do not express NOS (Moreno-López 2010), and, interestingly, l-NAME administration for 7 days in adult rats did not affect synaptic arrangement in the HN and HMNs (Sunico et al. 2005); (2) de novo expression of NOS-I in adult HMNs induced by axonal injury or by viral transduction of intact HMNs, both induced a change in excitatory/inhibitory balance on motor neurons that was reverted/avoided, at least in part, by interfering with NOS-I/NOS (Sunico et al. 2010; Montero et al. 2010; Moreno-López et al. 2011); and (3) in pre-symptomatic and early-symptomatic ALS mice, de novo expression of NOS occurs in motor neurons and glia concomitantly with an excitatory/inhibitory unbalance in HMNs which was partially restored/avoided by chronic treatment with l-NAME (Moreno-López et al. 2011; Sunico et al. 2011).

Mechanism of action by which endogenous NO controls synaptic dynamic during postnatal development could involve modulation of the actomyosin contraction apparatus. In this context, MLC phosphorylation triggers actomyosin contraction and neurite retraction (Luo 2000, 2002; Etienne-Manneville and Hall 2002). In this line, the small Rho GTPase RhoA and its major effector Rho kinase (ROCK) mediates fiber contraction by enhancing pMLC. RhoA/ROCK signaling, directly and/or indirectly activating MLC-kinase, phosphorylates MLC. It then induces actomyosin contraction and neurite outgrowth inhibition/retraction disturbing spine formation and maintenance. By decreasing synaptic connectivity during development, this mechanism has been proposed to underlie mental retardation (Newey et al. 2005). NO-dependent increase in pMLC levels has been reported in the HN and in the synaptic coverage of HMNs preceding NO-triggered synapse loss suffered by sick motor neurons (Sunico et al. 2010, 2011; Moreno-López et al. 2011). Here, increased levels of pMLC were measured in the HN at P18 and P21, however, chronic application of the NOS inhibitor avoided pMLC rise, thus supporting that NO could regulate actomyosin contraction during postnatal maturation of the HN. Whether pMLC increase occurs in synaptic structures before inputs remodeling and/or it is involved in NO-mediated regulation of HMNs morphology remain elusive.

HMNs participate in a number of functions such as respiration, chewing, sucking, swallowing, and phonation. Then, the HN receives afferents from pre-motor nuclei coding for oro-facial functions, thus involving many other structures coding respiratory-unrelated activity (Travers and Norgren 1983; Borke et al. 1983; Takada et al. 1984; Ugolini et al. 1987; Aldes 1990; Manaker et al. 1992). In this way, whether endogenous NO affects synaptic afferents from respiratory-related and/or -unrelated pre-motor structures is a matter that merit further investigation. This, together with subtle alterations in synaptic arrangement found after presumable partial inhibition (Moreno-López et al. 2004) of NOS-I in the HN, could explain why alterations in the breathing pattern of l-NAME-treated pups were not evidenced. In addition, the emergence of still to discover compensatory mechanisms in response to chronic inhibition of NOS cannot be discarded.

Administration route in our experiments ensures that l-NAME can affect most brain structures that express NOS. For example, transient expression of NOS during postnatal development includes brain structures such as cerebral cortex (Vercelli et al. 1999; Imura et al. 2005) and hippocampus (Chung et al. 2004), lateral geniculate nucleus (Cramer et al. 1995) and superior colliculus (Mize et al. 1997), cerebellum (Li et al. 1997), and olfactory bulb (Samama and Boehm 1996), among others. In this context, NO is believed to act as a retrograde messenger to stabilize synaptic connections via an activity-dependent mechanism (Williams et al. 1994). Therefore, it is possible that l-NAME infusion from P7 to P21 might alter networking in these structures, subsequently leading to some distortion in the correct acquisition of relevant functions such as visual processing, smell, learning and memory, or other associative and higher level tasks.

Dysregulation of NO synthesis and/or NOS expression has been suggested to lie behind synaptic dysfunction associated with cognitive decline in many neurological conditions (Moreno-López et al. 2011). At the neonatal stage, transient expression of NOS in HMNs seems to be essential for normal maturation of synaptic arrangement in the HN. Interestingly, HMNs development impairment is related with sudden infant death syndrome (Konrat et al. 1992; Lavezzi et al. 2010) and obstructive sleep apnea (Remmers et al. 1978). Normal anatomical maturation of HMNs (Cameron and Nunez-Abades 2000; Carrascal et al. 2005, 2015; Kanjhan et al. 2016a, b; Williams et al. 2019a, b) might be impaired by disruption of NOS transient expression in motor neurons. It can contribute to abnormal HN size, hypoplasia, and immaturity of HMNs found in sudden infant death syndrome (Konrat et al. 1992; O’Kusky and Norman 1995; Ottaviani et al. 2006; Lavezzi et al. 2010). In contrast, since NO over-production is cytotoxic (Moreno-López 2010; Moreno-López et al. 2011), transitory over-expression of NOS might affect HMN viability. In this context, the number of apoptotic cells in the HN was enhanced in sudden unexpected death in infancy (Ambrose et al. 2018). Furthermore, dysregulation of NOS expression could be involved in synaptic alterations in the HN observed in sudden infant death syndrome (O’Kusky and Norman 1995). At the electrophysiological level, developmental changes in passive and active membrane properties determine mature HMNs excitability (Cameron and Nunez-Abades 2000; Carrascal et al. 2005, 2015; Greer and Funk 2005). Thus, input resistance and rheobase (minimal current to elicit an action potential) decrease, by at least developmental increase in the expression of a “leak” potassium current mediated by TASK1 channels (Talley et al. 2000; Greer and Funk 2005). In addition, inward rectification current (*I*_h_) up-regulates during postnatal development. Interestingly, NO increases HMNs excitability by facilitating *I*_h_ current, and enhances input resistance and reduces rheobase (González-Forero et al. 2007; Montero et al. 2008) by at least inhibiting and/or impairing plasma membrane insertion of TASK1 channels (González-Forero et al. 2007; García-Morales et al. 2019). Therefore, dysregulation of NOS expression during this key period could lie behind HMN malfunctioning contributing to sudden infant death. Further, under distortion of transitory expression of NOS, the ratio excitatory/inhibitory inputs might be reduced leading to hypoactive HMNs after maturation which could enhance the probability to suffer obstructive sleep apnea. Accordingly, hypoglossal nerve stimulation is proposed as an effective long-term therapy in selected patients of this pathology (Kent et al. 2019; Gottlieb and Punjabi 2020). Altogether, it would be interesting to investigate whether disturbance of transient expression of NOS is a feasible etiopathogenic event underlying these pathologies.

## Data Availability

The datasets generated and/or analyzed during the current study are available from the corresponding authors on reasonable request.
